# Lycorine is a novel inhibitor of the growth and metastasis of hormone-refractory prostate cancer

**DOI:** 10.18632/oncotarget.3610

**Published:** 2015-04-12

**Authors:** Meichun Hu, Shihong Peng, Yundong He, Min Qin, Xiaonan Cong, Yajing Xing, Mingyao Liu, Zhengfang Yi

**Affiliations:** ^1^ Shanghai Key Laboratory of Regulatory Biology, Institute of Biomedical Sciences and School of Life Sciences, East China Normal University, Shanghai 200241, China; ^2^ Institute of Bioscience and Technology, Texas A&M University Health Science Center, Houston, Texas 77030, USA

**Keywords:** Lycorine, hormone-refractory PCa, STAT3 signaling, tumor growth and metastasis

## Abstract

Lycorine, a natural alkaloid extracted from the Amaryllidaceae plant family, has been reported to exhibit a wide range of physiological effects, including the potential effect against cancer. However, the anti-prostate cancer (PCa) efficacy of Lycorine remains unrevealed. In this context, we figured out Lycorine's anti-proliferative and anti-migratory properties for PCa treatment. Lycorine inhibited proliferation of various PCa cell lines, induced cell apoptosis and cell death. Here we showed that Lycorine decreased proliferation, migration, invasion, survival and EMT of prostate cancer cell lines. Subcutaneous and orthotopic xenotransplantations by ectopic implantation of the human hormone-refractory PC-3M-luc cells were used to confirm *in vivo* anticancer effects of Lycorine. Lycorine inhibited both growth and metastasis in multiple organs (liver, lung, kidney, spleen and bone) *in vivo* and improved mice survival. Lycorine prevented EGF-induced JAK/STAT signaling. Importantly, anti-cancer effects of Lycorine were dependent on STAT expression. We suggest that Lycorine is a potential therapeutic in prostate cancer.

## INTRODUCTION

Prostate cancer (PCa) is the principle malignant tumor threatening the health of senile male all over the world. According to the latest statistics, PCa accounts for 27% of all cancer incidences and ranks the second leading cause of cancer-related mortality among men in the US [1]. PCa first manifests as an androgen-dependent (AD) disease and can be treated with androgen deprivation therapy, but it will eventually progresses from AD to androgen independent (AI). The hormone-refractory PCa is the end stage of AI and causes the majority of PCa patient deaths [2]. It is estimated that by January 1, 2024, the whole cancer survivors in American will increase to nearly 19 million and PCa will still be the most prevalent cancer among males. The massive survivorship indicates that the treatment of PCa is an encouraging cancer research direction [3]. With the increasing incidence, mortality, and survivorship, there is an urgent need for novel drugs that can radically cure PCa, especially the hormone-refractory PCa. Lycorine, a medicinal plant-derived phenanthridine Amaryllidaceae alkaloid [4], seems to possess such properties for PCa drug research and development.

Lycorine is a pyrrolo[*de*]phenanthridine ring-type alkaloid extracted from Amaryllidaceae genera and possesses various biological effects including anti-tumor [5], antiviral [6], antimalarial [7] and antiinflammation [8]. Although Lycorine does not have a defined target or mechanism of action, it is a candidate anti-inflammatory and anti-cancer drug. For instance, the drug, which contains Lycorine as effective component, has been clinically used in Russian as an expectorant to treat chronic and acute inflammatory processes in lungs and bronchial diseases [9]. Recently, a new function of Lycorine in promoting hematopoietic stem and progenitor cell (HSPC) niche colonization has been reported. Lycorine increases HSPC lodgement during development and ultimately leads to a sustained increase in the size of the stem cell pool into adulthood [10]. Several studies have shown that Lycorine exhibits anti-cancer activities. For example, it has selective cytotoxicity effects on leukemia, cervical cancer and multiple myeloma [11–16]. However, so far, there is no research about Lycorine's effects in PCa. Accumulating evidences indicate that abnormalities in JAK/STAT pathway are involved in the oncogenesis of several cancers [17]. Many researches confirm STAT3 as a causal role in oncogenesis, and provide validation of STAT3 as an oncotarget for cancer drug discovery [18]. Also, several studies propose that abnormalities in STAT3 pathway causes the oncogenesis of PCa [19]. Furthermore, recent studies identify STAT3 as a direct transcriptional activator of Twist, which is one of the most important drivers promoting epithelial mesenchymal transition (EMT) [20]. Moreover, the activation of epidermal growth factor receptor (EGFR) signaling pathway induces cancer cell EMT via STAT3-mediated Twist gene expression. Epidermal growth factor (EGF) increases Twist transcripts and protein in EGFR-expressing lines. Continuously activated STAT3 significantly promotes Twist transcription. EGFR cooperates with STAT3 to induce EMT in cancer cells via increasing Twist gene expression [21]. Overall, the impairment of STAT3 activation can inhibit tumor growth and metastasis. Therefore, targeting STAT3 is a valid strategy for cancer therapy [22]. Currently, some STAT3 inhibitors, including small molecular compounds, peptides, and oligonucleotides, have been developed to combat PCa, and it is rational to anticipate their potential therapeutic value in clinic [23–25].

In our study, we demonstrate that Lycorine is a novel inhibitor of the growth and metastasis of hormone-refractory PCa. We present that Lycorine inhibits proliferation, migration, invasion and survival of various PCa cell lines, induces cell apoptosis and cell death, and reverses EMT process of prostate cancer cell lines. *In vivo* experiments show that intraperitoneal (i.p.) administration of Lycorine reduces both weight and volume of ectopically PC-3M subcutaneous xenografts by about 80% while exhibiting no obvious toxicity. Lycorine also inhibits PCa growth and metastasis when tested in the PC-3M-luc orthotopic xenograft model. Lycorine inhibits the activation of EGF induced JAK/STAT signaling and multiple STAT3 downstream targets, such as cyclin D1, Bcl-2, Bcl-xL, matrix metalloproteinase 2 (MMP2), and the EMT promoter Twist. Importantly, these anti-cancer effects of Lycorine are dependent on STAT3 expression. In conclusion, our findings suggest that Lycorine is a potential therapeutic in prostate cancer.

## RESULTS

### Lycorine inhibits proliferation, migration and invasion in PCa cells

**Figure 1:**
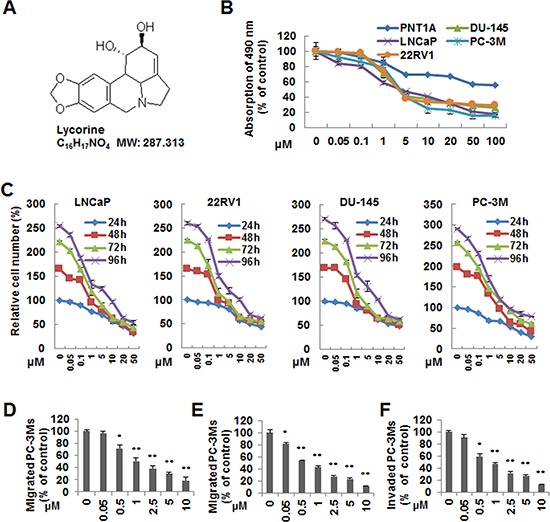
Effects of Lycorine on proliferation, migration and invasion of PCa cells *in vitro* **A.** Chemical structure of Lycorine. **B.** PC-3M, LNCaP, 22RV1, DU145 and PNT1A were treated with indicated concentrations of Lycorine (0, 0.05, 0.1, 1, 5, 10, 20, 50 and 100 μM) for 48 hours. Cell viability was assessed by MTS assay (*n* = 3). **C.** PC-3M, LNCaP, 22RV1 and DU145 cells were treated with Lycorine with indicated concentrations (from 0 μM to 50 μM) and hours (from 24 h to 96 h) to test the time- and dose-dependent effects. Cell viability was assessed by MTS assay (*n* = 3). **D.** PC-3M cells were allowed to migrate cross a “wound” when treated with Lycorine (from 0 μM to 10 μM) for 12 hours. The number of migrated cells were calculated. **E.** PC-3M cells were seeded on the upper chamber of Transwell. After 5 to 7 hours incubation with Lycorine (from 0 μM to 10 μM), migrated cells were fixed, stained and counted. **F.** PC-3M cells were treated with Lycorine (from 0 μM to 10 μM) for 12 hours and seeded in the upper chamber of Transwell coated with Matrigel to invade for another 12 hours. Invaded cells were fixed, stained and counted. All data are represented as mean ± S.D. from triplicate wells. *p < 0.05, **p < 0.01, ***p < 0.001, as compared to control.

Tumor malignancy relies on its ability of growth and metastasis without control. To investigate the anti-cancer activity of Lycorine on PCa, especially the hormone-refractory PCa, 4 typical malignant hormone-refractory PCa cell lines, PC-3M, DU145, LNCaP and 22RV1, as well as a human normal prostate epithelium immortalized cell line PNT1A, were subjected to the MTS assay. Fig. 1A showed the chemical structure of Lycorine. As shown in Fig. 1B, Lycorine inhibited cell proliferation in a dose-dependent manner in the abovementioned 4 PCa cell lines, and the IC50 ranged from 5 μM to 10 μM. Fig. 1B also showed Lycorine had little effects on PNT1A cell's proliferation. Collectively, Lycorine had appreciable selectivity between normal human epithelial cells and cancer cells. Furthermore, Fig. 1C illustrated that Lycorine inhibited PCa proliferation in a time- and dose-dependent manner on these 4 PCa cell lines. To determine the effects of Lycorine on PCa metastasis, we performed cell migration and invasion assays using cell line PC-3M with highly malignant mobility. Lycorine, in a dose-dependent manner, significantly inhibited PC-3M cell wound healing (Fig. 1D), migration (Fig. 1E), and invasion (Fig. 1F).

### Lycorine retards PCa cell growth *in vitro* through inducing apoptosis

**Figure 2:**
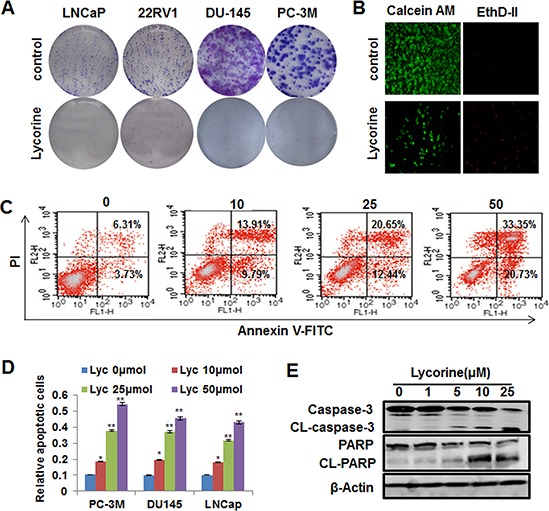
Effects of Lycorine on PCa cell colony formation and apoptosis **A.** 4 PCa cells were treated with different concentrations of Lycorine (from 0 μM to 10 μM), respectively. On the 8th day, colonies were fixed, stained, and counted. Representative results of these 4 cell lines were taken by camera. **B.** PC-3M cells were treated with different concentrations of Lycorine (from 0 μM to 50 μM) for 48 h and stained with live/dead reagent. The cell-permeable fluorescent dye Calcein AM stains live cells (green) and the dead cells are stained by EthD-II (red). Stained live and dead cells are visualized by fluorescence microscopy. **C.** PC-3M cells were treated with indicated concentrations of Lycorine (from 0 μM to 50 μM) for 48 hours. Apoptosis was assessed by Annexin V/PI staining and flow cytometry (*n* = 3). **D.** Statistic result of apoptosis results of PC-3M, DU145 and LNCaP cells when treated with indicated concentrations of Lycorine (from 0 μM to 50 μM) for 48 hours. **E.** PC-3M cells were incubated with various concentrations of Lycorine (from 0 μM to 25 μM) for 48 hours. Effects on the expression of CL–caspase 3 and PARP were determined by Western blotting. Beta-actin served as a loading control.

To further determine the function of Lycorine's anti-proliferation activity to PCa cells, the colony formation assay was conducted. Results showed that Lycorine (5 μM) significantly inhibited 4 PCa cell lines, PC-3M, DU145, LNCaP and 22RV1's colony formation (Fig. 2A). Statistical results of these 4 cell lines’ colony formation under the treatment of Lycorine were shown in Supplementary Fig. S1A. In addition, the live/dead staining was used to test Lycorine's toxicity to PCa cells. As shown in Fig. 2B, Lycorine potentiated PC-3M cell death. Lycorine did not induce cell-cycle arrest (Supplementary Fig. S1B, left) and the statistical result showed no significant difference between the cell-cycle distributions (Supplementary Fig. S1B, right), but Lycorine caused a dose-dependent induction of apoptosis in PC-3M cells. Apoptotic cells raised from 10.04% to 54.08% with the Lycorine concentration increased from 0 μM to 50 μM after 48-hour treatment (Fig. 2C). Similarly, Lycorine also induced cell apoptosis in DU145 and LNCaP cells (data not shown) and Fig. 2D illustrated the statistical results of Lycorine's apoptotic effects to 3 PCa cells. And clear cleavages of PARP and caspase-3 occurred when treated with Lycorine (Fig. 2E). All these results suggested that Lycorine suppressed PCa cell growth through its proapoptotic effects.

### Lycorine inhibits PC-3M subcutaneous tumor growth *in vivo*

**Figure 3:**
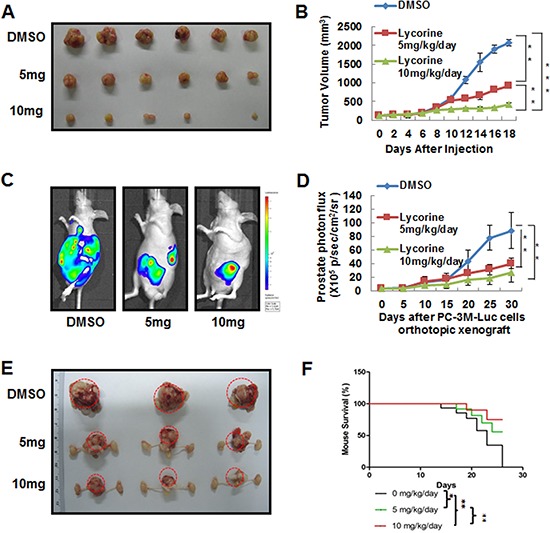
*In vivo* anticancer effects of Lycorine on PCa mouse xenograft models **A.** Representative images of PC-3M subcutaneous tumor xenografts after mice sacrificed. PC-3M cells were injected subcutaneously into the nude mice. The tumor model was established according to the steps described in Materials and Methods. **B.** Quantitative analysis of growing tumor volume in mice back subcutaneous every 2 days. **C.** Representative *in vivo* bioluminescence images of mice bearing orthotropic–injected PC-3M-luc cells. **D.** Quantization of whole-body bioluminescence (total photon flux) in control and treatment groups. **E.** Representative images of prostate of mice bearing orthotropic–injected PC-3M-luc cells after mice sacrificed. **F.** Graphic representation of mice survival curves during therapeutic administration. The means and 95% confidence intervals (error bars) were presented (***P < 0.001, **P < 0.01, *P < 0.05).

PC-3M cells were injected into nude mice to establish the PCa subcutaneous tumor xenograft model. Mice were divided into 3 groups (*n* = 10 per group) and treated with Lycorine at 5 mg/kg/day or 10 mg/kg/day or vehicle control. At the day 18, mice were sacrificed; tumor xenografts were dissected (Fig. 3A) and the tumor weights were calculated in Supplementary Fig. S1C. From Fig. S1C it could be concluded that Lycorine significantly suppressed the tumor growth of PCa. The average tumor volume of control group was 2154 ± 119 mm^3^, whereas tumor volume in Lycorine-treated group was 989 ± 32 mm^3^ for 5 mg/kg/day group and 478 ± 47 mm^3^ for 10 mg/kg/day group, respectively. And statistical result showed significant difference between the drug-treated groups and the control group (Fig. 3B).

### Lycorine inhibits PC-3M orthotopic tumor growth and metastasis *in vivo*

**Figure 4:**
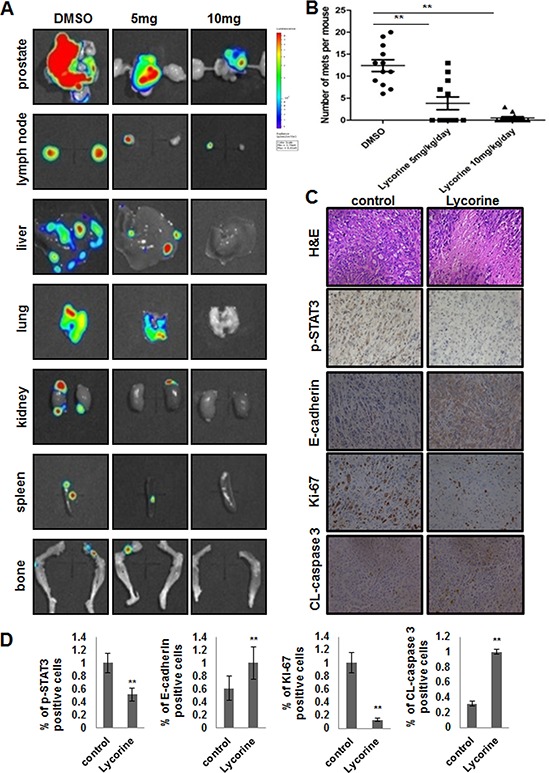
Effects of Lycorine on PCa metastasis *in vivo* **A.** Representative photos of metastasis site (prostate, lymph node, liver, lung, kidney, spleen, bone) monitored by *in vivo* bioluminescence detection of PC-3M-luc cells in excised organ from control and treatment tumor-bearing nude mice. **B.** Survey of mets to the whole body of each mouse monitored by *in vivo* bioluminescence detection of PC-3M-luc cells in excised organ from control and treatment tumor-bearing nude mice. **C.** Primary tumors sections were excised and processed for H&E staining and immunohistochemical analysis to detect Ki-67, cleaved-caspase 3, p-STAT3 and E-cadherin. **D.** Quantitative analysis of Ki-67, cleaved-caspase 3, p-STAT3 and E-cadherin about the immunohistochemistry results. The means and 95% confidence intervals (error bars) were presented (***P < 0.001, **P < 0.01, *P < 0.05).

The orthotopic transplantation tumor model is widely used to imitate the real clinical situation of cancer progression in drug research. In an attempt to mimic human disease to the maximum extent, we evaluated Lycorine's chemotherapeutic potential on PC-3M orthotopic tumor growth and metastasis model *in vivo*. Briefly, a luciferase-expressing PC-3M cell line (PC-3M-luc) was established by stably transfected with luciferase-expressing plasmids. After injected orthotopicly into the mice dorso lateral prostate from day 0 to day 30, PC-3M-luc cells exhibited bioluminescence which could be traced by photon flux indexes to represent the tumor sizes using the IVIS 2000 Luminal Imager system. Mice were divided into 3 groups (*n* = 20 per group) and treated with Lycorine at 5 mg/kg/day or 10 mg/kg/day or vehicle control. Tumors in the whole body of each mouse were imaged by IVIS every 5 days to determine local tumor growth and tumor cells dissemination. As shown in Fig. 3C, Lycorine evidently impaired the PC-3M-luc orthotopic xenografts in tumor-bearing mice. In the control group, bioluminescence was detected in the whole parts of mice abdomen and increased remarkably with day number increase (Fig. 3D). Treatment with Lycorine statistically reduced the photo flux indexes (Fig. 3D). Administration of 10 mg/kg/day of Lycorine almost completely blocked tumor growth. As Fig. 3E showed, at the day 30 the nude mice were sacrificed and the orthotopic xenografts were stripped for photograph. The average normalized photon flux of the 5 mg/kg/day Lycorine treated group and 10 mg/kg/day Lycorine treated group was (41 ± 2.34) × 10^5^p/sec/cm^2^/sr and (23 ± 4.01) × 10^5^p/sec/cm^2^/sr, respectively, while that of vehicle control group was (82 ± 5.78) × 10^5^p/sec/cm^2^/sr (Fig. 3D). Supplementary Fig. S1D showed the statistical result of final volumes of orthotopic xenografts. At day 30, for the whole 20 mice in each group, 6 mice died in the control group, while the number was 4 and 2 for the 5 mg/kg/day Lycorine treated group and 10 mg/kg/day Lycorine treated group, respectively (Fig. 3F). When referring to Lycorine's biosafety, treatment of Lycorine at the given concentration had little effect on mice body weights (Fig. S1E), and this finding confirmed to previous published results that Lycorine had low toxicity to mouse at the curative dose [16]. The anatomy results of sacrificed mice in the 10 mg/kg/day group showed no obvious pathological change on main organs, when compared to the control group (Fig. S1F). In addition, Lycorine inhibited PCa tumor metastasis in the orthotopic tumor growth and metastasis model. After dissection, photo signals were detected in typical metastatic target organs (liver, lung, kidney, spleen and bone), as well as orthotopic prostate and lymph node (Fig. 4A). And Lycorine significantly decreased metastatic nodes numbers in target organs (Fig. 4B). Together, our data indicated that administration of Lycorine therapeutically blocked PCa tumor growth and metastasis.

Interestingly, when we explored the growth and metastasis related signal pathways by H&E staining and immunohistochemistry analysis, we found the expression of E-cadherin and CL-caspase 3 increased, while the expression of p-STAT3 and Ki-67 decreased in Lycorine-injected mice tumors in a dose of 10 mg/kg/day, compared to the control group (Fig. 4C). This finding suggested that Lycorine might suppress the STAT3 pathway and have relationship with the reversal of EMT *in vivo*. Fig. 4D exhibited the statistical results of these four tumor progression markers. In summary, these *in vivo* immunohistochemistry results are in agreement with our *in vitro* results and indicate that Lycorine therapeutically suppresses PCa tumor growth and metastasis in orthotopic xenograft model.

### Lycorine reverses EGF-induced EMT in PCa cells

**Figure 5:**
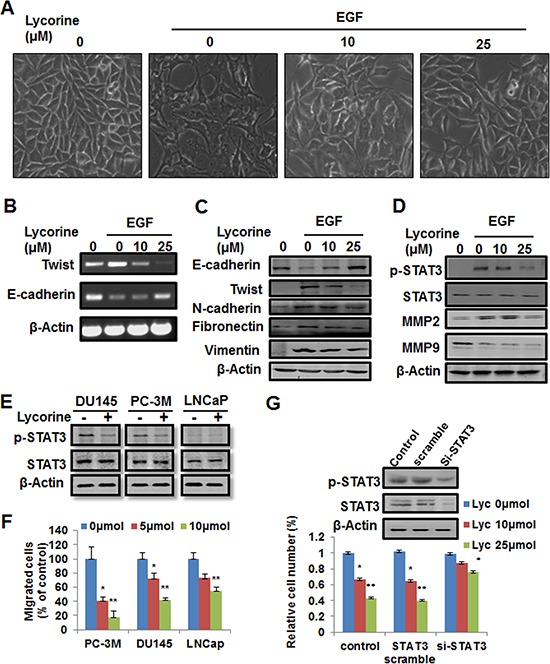
Reverse effect of Lycorine on EMT in PCa cells **A.** PC-3M cells were treated with 10ng/ml EGF and indicated concentrations of Lycorine (0, 10 and 25 μM) for 48 hours, and then the cell morphology changes were photographed. **B–C.** RT-PCR analysis and Western blotting analysis of Twist, E-cadherin, N-cadherin, vimentin and fibronectin in PC-3M cells. Cells were treated with 10ng/ml EGF and indicated concentrations of Lycorine (0, 10 and 25 μM) for 48 hours. Cell lysates were subjected to RT-PCR analysis (B) and Western blotting analysis (C). Beta-actin served as a loading control. **D.** Western blotting analysis for p-STAT3, STAT3, MMP2 and MMP9 in PC-3M cells. Cells were treated with10ng/ml EGF and indicated concentrations of Lycorine (0, 10 and 25 μM) for 48 hours, and then the cells were subjected to Western blotting analysis. Beta-actin served as a loading control. **E.** Lycorine's effects on the expression levels of endogenous STAT & p-STAT3 in DU145, PC-3M and LNCaP cells. Cells were treated with or without 10 μM Lycorine for 48 hours, and then the cells were subjected to Western blotting analysis. **F.** Statistic result of Lycorine's effects on the migration of DU145, PC-3M and LNCaP cells. The Transwell migration assay was conducted as described in Materials and Methods. **G.** PC-3M cells were transiently transfected with STAT3-siRNA or the non-targeting scrambled siRNA for 48 hours and the interference effect of STAT3 was detected by Western blotting. Cells were treated with Lycorine (0, 10 and 25 μM) for another 48 hours and cell viability was tested by MTS assay.

Tumor cell migration and invasion are dependent on epithelial cells increasing their ability to migrate through a process named EMT, and EMT is strongly induced by EGF signaling in PCa and other cancer types [26–29]. As one of the most important EMT markers and a master regulator of morphogenesis, Twist plays an essential role in tumor metastasis, and can be considered as an oncotarget [30]. Previous reports have revealed that EGF directly stimulates Twist transcription [21, 23] and Twist is a direct target gene of STAT3 [31]. Therefore, we examined whether Lycorine had inhibitory effects on EGF-induced EMT. After exposed to EGF, PC-3M cells became polar and polygen, from the pebble shape or fusiform to multiple antenna in morphology (Fig. 5A), decreased the expression of an epithelial cell marker (E-cadherin), and increased the expression of mesenchymal cell markers (N-cadherin, vimentin and fibronectin) (Fig. 5C). In contrast, cells exposed to Lycorine had a dose-dependent decrease in expression of N-cadherin, vimentin and fibronectin and a corresponding increase in E-cadherin expression (Fig. 5C) and the morphology change had been largely reversed to the epithelial cell like stage (Fig. 5A). Because in previous studies STAT3 decreased E-cadherin expression and stimulated EMT by repression of the transcriptional factor Twist, we have assessed both protein and RNA levels of Twist in PC-3M cells. We observed a significant decrease in Twist mRNA level in response to Lycorine (Fig. 5B). This result was consistent with the increased E-cadherin expression (Fig. 5C). Our results clearly demonstrate that Lycorine promotes the epithelial cell characteristics and suppresses mesenchymal features in PC-3M cells. RT-PCR result (Fig. 5B) confirmed that EGF-induced EMT was significantly blocked by Lycorine through inhibiting the expression of Twist, whereas N-cadherin, vimentin and fibronectin levels were decreased by Lycorine (Fig. 5B and 5C). Because Lycorine caused EMT reversal in PC-3M cells, it was reasonable to expect a decreased migration potential as well. Our data showed that Lycorine significantly suppressed the expression of migration markers, MMP2 and MMP9, as well as the activated STAT3 levels under the stimulation of EGF (Fig. 5D). All these results suggest that Lycorine suppresses EGF-induced EMT.

### Lycorine's anti-cancer effects are dependent on STAT3 expression

To confirm whether Lycorine's suppression in EGF-induced EMT is directly mediated by inhibiting STAT3, we used the 3 cell line models, DU145, LNCaP, PC-3M, that expressed different amounts of STAT3 and p-STAT3 (Fig. 5E), and tested their response to Lycorine. Fig. 5F showed that in PC-3M cell, which had the most amount of STAT3/p-STAT3 level, exhibited the largest response to Lycorine in the migration assay. Using a standard procedure to measure cell migration, we observed a significant decrease in the percentage of migrated cells due to STAT3 expression as compared between these 3 cell lines (Fig. 5F). For DU145 and LNCaP cells, although their migration were also abrogated by Lycorine, the inhibition effect was less than that in PC-3M. Besides, for further investigation whether STAT3 is involved in Lycorine's inhibition in PCa cells growth and metastasis, PC-3M cells were treated with 10 μM or 25 μM Lycorine for 48 hours following transient transfection with STAT3 siRNA, and Western blotting was performed (Fig. 5G). PC-3Ms transfected with STAT3 siRNA displayed reduced levels of endogenous STAT3 expression (Fig. 5G, upper). Lycorine induced a dramatic decrease in cell viability in STAT3 high-expression cells transfected with control or STAT3 sramble siRNA, whereas no significant decrease of cell viability was observed in STAT3 knockdown cells (Fig. 5G, bottom). Taken together, all these data indicate that STAT3 is involved in Lycorine induced inhibition to PCa cells and the anti-cancer effects of Lycorine are dependent on STAT3 expression.

### Lycorine suppresses STAT3 transcriptional activity in PCa cells

**Figure 6:**
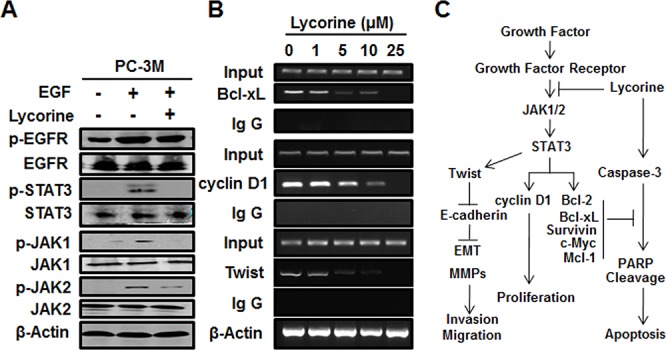
Effect of Lycorine on JAK/STAT3 pathway in PCa cells **A.** PC-3M cells were treated with 10ng/ml EGF and 10 μM Lycorine for 24 hours, then prepared for Western blotting of the expression of EGFR, JAK1/2, STAT3 and their phosphorylation. **B.** PC-3M cells were treated with increasing concentrations of Lycorine (from 0 μM to 25 μM) for 24 hours before being applied to ChIP assay to test the STAT3-dependent transcriptional activity of Bcl-xL, cyclin D1, and Twist. Beta-actin was used as internal controls. **C.** Schematic model of the hypothesized mechanism by which Lycorine inhibits PCa growth and metastasis.

It's well-known that activation of STAT3 culminates in the transcription of its target genes and that EGF signaling relevant to tumor growth and metastasis is partially mediated by JAK1/2 phosphorylation. Therefore, we tested the action of Lycorine on these critical receptor tyrosine kinases. We found that Lycorine significantly inhibited the phosphorylation of STAT3 (Tyr705) under the stimulation of EGF (Fig. 6A). And such inhibitory action was in parallel with a rapid dephosphorylation of upstream kinases of STAT3, including JAK1 (Tyr1, 022/1, 023) and JAK2 (Tyr1, 007/1, 008) (Fig. 6A). STAT3 translocation to the nucleus results in a specific DNA binding that in turn increases target gene transcription; we next determined whether Lycorine suppressed DNA-binding activity of STAT3 by chromatin immunoprecipitation (ChIP) assay. Our results showed that Lycorine decreased STAT3 DNA-binding activity to its typical target genes such as Bcl-xL, cyclin D1 and Twist in a concentration-dependent manner, with effective concentrations around 10 μM (Fig. 6B). Lycorine effectively regressed STAT3's translocation from cytoplasm to nuclei under the stimulation of EGF (Supplementary Fig. S2A). Q-PCR analysis confirmed that STAT3 target genes were down-regulated by Lycorine (Supplementary Fig. S2B and S2C). To further address such a possibility, we examined the inhibitory effect of Lycorine on STAT3 in its target genes’ protein levels and similar results were observed (Supplementary Fig. S2D). In summary, as Fig. 6C sketched, all these results prove that Lycorine blocks tumor growth and metastasis by suppressing STAT3 transcriptional activity in PCa cells.

## DISCUSSION

Men with hormone-refractory PCa are at high risk for developing distant metastasis, which results in clinically significant disability and mortality [32]. Although great therapeutic advances in hormone-refractory PCa has been made, it's still inevitable came along with the treatment failure [33]. Chemotherapy is useful currently in metastatic castrate-resistant PCa. The key drugs are docetaxel and cabazitaxel since two randomized trials have proven their efficacy [34]. Several agents, such as selenium, lycopene, soy products, green tea, pomegranate phenolics, apigenin, and vitamins D and E, are effective in the prevention of PCa [35–38]. However, there is still a limited number of agents that are effective and selective in the prevention and/or treatment of late-stage hormone-refractory PCa. Our principle findings in this study show that the anti-tumor activity of Lycorine toward PCa at physiologically achievable concentrations is moderate. We present that Lycorine induces apoptosis and inhibits migration and invasion of hormone-refractory PCa cells (Fig. 1). Administration of Lycorine delays tumor growth and metastasis and reduces both tumor weight and volume and distant metastasis (Fig. 3 and 4). In addition, Lycorine abrogates the expression of p-STAT3 (Fig. 4C). All these results provide a characterization of the unique anti-tumor properties of Lycorine as a potential candidate for the treatment of hormone-refractory PCa.

Another significant result in this study is that Lycorine reverses EMT through STAT3 mediated Twist decrease, thus exhibits a novel mechanism that Lycorine inhibits cancer metastasis. PCa cells in metastasized tumors are known to display both epithelial and mesenchymal phenotypes including morphology and gene expression profiles, and metastatic prostate tumors are likely comprised of heterogeneous populations of both epithelial and mesenchymal cells. With regard to the mechanism of interconversion between epithelial and mesenchymal phenotypes through EMT and MET, it is interesting to notice the induction by growth factor engagement [39]. Previous reports imply EGFR signaling are implicated in PCa progression and EGF promotes disassembly of cell–cell junctions, decrease of E-cadherin and increase of Twist. These effects are dependent on activation of STAT3 as a mediator of EGF/EGFR-driven cell migration and EMT. Knockdown of endogenous STAT3 also prevents EGFR-mediated EMT and cell migration [40]. Additionally, some reports declaimed that targeting PCa cell EMT could be an attractive new therapeutic direction for the inhibition of PCa [41]. Our research for the first time uncovered this links between Lycorine, STAT3 and EMT. Because many types of cancer cells express STAT3 and EMT is a typical phenomenon upon metastatic stimuli, such as growth factor, Lycorine may have functions in inhibiting other tumor metastasis *via* the same mechanism that revealed in our research.

Lycorine has been suggested to exert its anti-tumor activity in leukemia and in solid tumors through pro-apoptotic effects [11–14]. In this study, Lycorine provides significant therapeutic benefit in mice bearing grafts of the PC-3M model at non-toxic doses. Thus, Lycorine may be an excellent candidate to combat PCa. Besides, a previous report declares Lycorine is able to cross the blood brain barrier and induces no CYP3A4 inhibitory activity [42]. All of these properties make Lycorine potential for clinic application. Indeed, treatment with Lycorine inhibited tumor xenograft growth and metastasis and increased the overall health and survival of nude mice implanted with PC-3M-luc cells. However, these effects were measured in tumors obtained from only one cancer cell lines. To better utilize Lycorine's therapeutic potential, further investigations are needed to be performed on other types of xenografts using different PCa cell lines. Furthermore, researches to determine Lycorine's underlying mechanisms besides STAT3 in PCa are warranted.

Conclusively, this study is the first research to examine Lycorine's anti-cancer mechanistic role of JAK/STAT3 signaling in PCa tumor growth and metastasis and identify a perturbance in a plethora of JAK/STAT signaling proteins in PCa. Our results support Lycorine as a potent candidate for drug repositioning with advantages of toxicity and pharmacokinetics profiles already known as well as time-and cost-saving. To finish, our study proves evidences that STAT3 is a potential therapeutic target for PCa treatment and strengthens the possibility of Lycorine as a natural product drug in anticancer therapy and shows promise for future clinical translation.

## MATERIALS AND METHODS

### Cell culture, animals and reagents

Hormone-refractory PCa cell line PC-3M, LNCaP, 22RV1 and DU145 were purchased from ATCC. PNT1A (a human normal prostate epithelium immortalized cell line) was a kind gift from Professor Hanyi Zhuang of Shanghai Jiaotong University. PC-3M cells and DU145 cells were cultured in Dulbecco's Modified Eagle Medium (Gibco). PNT1A, 22RV1 and LNCaP cells were maintained in RPMI-1640 medium (Gibco). Both mediums were supplemented with 10% fetal bovine serum (Wisent). In addition, PC-3M cells were transfected with pGL4 vector (Promega) which stably expressed luciferase and selected in G418 to screen the stable PC-3M-luc cell line. All cells were incubated at 37°C of 5% humidified CO_2_. Mice were obtained from National Rodent Laboratory Animal Resources, Shanghai Branch of China. All animal experimental protocols were approved by the Animal Investigation Committee of the Institute of Biomedical Sciences, East China Normal University. Lycorine (purity >98%) was purchased from Shanghai Winherb Medical Science. A 50 mM stock solution was prepared in dimethyl sulfoxide (DMSO; Sigma), stored at −20°C and diluted as needed in cell culture medium. Recombinant human EGF was obtained from BD Biosciences. Most appropriate antibodies were purchased from Cell Signaling Technology, unless otherwise specified.

### Cell viability assay

Cell viability of corresponding cells was determined by MTS assay according to previous methods [43]. PCa cells and PNT1A (5 × 10^3^ cells/well) were treated with various concentrations of Lycorine for 48 h or indicated time. The Aqueous One Solution (Promega) was used according to manufacturer instructions, and the absorption of 490 nm was measured. Three independent experiments with triplicate were carried out.

### Migration assay

The inhibition of tumor cell migration by Lycorine was determined by wound-healing migration assay and chamber migration assay. Briefly, tumor cells were allowed to grow into full confluence in 6-well plates, then the “wounds” were created by a sterile pipette tip. Fresh medium containing 10% FBS and various concentrations of Lycorine was added. After 12 h incubation at 37°C, cells were fixed with 3.7% paraformaldehyde and photographed. The migrated cells were manually quantified. The percentage inhibition of migrated cells was expressed using the untreated group as 100%. Besides, cell migration assay by Transwell/Boyden chambers (8 μm; BD Biosciences) was also conducted. Serum-starved PCa cells (5 × 10^4^ cells) in 100 μL medium with 0.5% FBS were pretreated with Lycorine (from 0 μM to 10 μM) for 30 minutes. Cells were then seeded on the upper chamber of Transwell and migrated to the lower chamber with 600 μL medium. After 5 to 7 hours incubation, non-migrated cells were removed with cotton swabs, and migrated cells were fixed with cold 3.7% paraformaldehyde and stained with 0.1% crystal violet. Images were taken with an inverted microscope (Olympus; magnification, × 100), and migrated cells in 4 random fields were quantified by manual counting. Three independent experiments with triplicate were carried out.

### Invasion assay

Invasion assay was also performed using Transwell/Boyden chambers coated with Matrigel as described previously [44]. In brief, after 12 hours pretreatment with indicated concentrations of Lycorine in 6-well plate, tumor cells were detached with trypsin and resuspended as signal cells. A total of 5 × 10^4^ cells in 100 μL of serum-free medium were added in the upper chamber, and 600 μL of complete medium were added at the bottom. Different concentrations of Lycorine were added to both chambers. The cells were allowed to invade for 12 h at 37°C. Invaded cells were stained with 0.1% crystal violet and counted manually. The percentage of inhibition was expressed with control as 100%.

### Tumor cell clonogenic assay

To mimic individual cell development into macroscopic cell clones, the cell clonogenic assay was performed as previously described [44]. Tumor cells were seeded into 6-well plate and allowed to grow for 24 h. Cells were then treated with different concentrations of Lycorine. On the 8th day, colonies were fixed in 3.7% paraformaldehyde, stained with 0.1% crystal violet and counted manually.

### Live/dead staining assay

The live or dead status of cells was determined by live/dead reagent (Invitrogen), which was used to measure intracellular esterase activity and plasma membrane integrity [45]. The cell-permeable green fluorescent dye Calcein AM (Ex/Em = 488/518 nm) is used to stain live cells. Dead cells can be easily stained by EthD-II, a cell non-permeable red fluorescent dye (Ex/Em = 488/615 nm). Stained live and dead cells can be visualized by fluorescence microscopy using a band-pass filter (detects FITC and rhodamine).

### Cell cycle analysis

Cell cycle analysis was conducted as described [44]. PCa cells were treated with Lycorine for 36 hours followed by PBS washes. Cells were then fixed with cold 70% ethanol at 4°C for at least 12 hours. PI working solution was added before flow cytometry analysis (FACS Calibur, BD biosciences).

### Apoptosis assay

Apoptosis was measured using the Apoptosis Detection Kit (BD Biosciences) as described [44]. PCa cells were treated with Lycorine for 48 hours then collected, washed, and stained with annexin V–FITC and PI for 15 minutes before evaluation by flow cytometry (FACS Calibur, BD biosciences).

### Western blotting

PCa cells were treated with Lycorine for indicated time and concentration; then lysed in RIPA buffer. Primary tumors were well minced, and also lysed with RIPA buffer. Protein concentration was determined using a Bicinchoninic acid assay (Thermo Scientific). Protein samples were run on 8% to 12% SDS-PAGE gels and transferred to polyvinylidene difluoride membranes (Gibco). The membranes were incubated overnight using specific antibodies. The signals were visualized via the Odyssey Western blotting detection system.

### Immunofluorescence staining assay

For immunofluorescence staining of tumor cells, human PCa cells were plated onto gelatin-coated cover slips. After treatment with or without Lycorine (as indicated in the figures) for 24 hrs, cells were fixed with 3.7% paraformaldehyde for 15 min at room temperature, permeabilized with 0.1% Triton X-100 for 5 min, and then blocked with 1% BSA for 1 hr. Cells were incubated with STAT3 antibody (as indicated in the figures) overnight. After washing with PBS, cells were incubated with the appropriate secondary antibody for 1 hr. 4,6-diamidino-2-phenylindole (DAPI) was further used to stain nucleus. Photographs were obtained with a Leica Confocal microscope.

### Real time PCR

RNA was reverse transcribed with miScript II RT Kit (Qiagen, Valencia, CA) following the manufacturer's instructions. The resulting cDNA was used for real time PCR analysis. For individual mRNA quantification we used miScript Primer Assays. For polymerase chain reaction (PCR) analysis, the reactions were performed in a Thermal iCycler (Biorad, Hercules, CA). The primers and probes for Bcl-2, Bax, cyclin D1, β-Actin [45], E-cadherin and Twist [31] were used as previously described. All primers were synthesized by Invitrogen and checked for specificity before use.

### Quantitative real-time PCR

The RNA isolation and quantitative real-time PCR were performed as described previously [46]. Cells were harvested by brief centrifugation directly after cell sorting or after treatment with Lycorine, and pellets were stored for RNA isolation. Primers sequences were as previously reported [45]. All primers were synthesized by Invitrogen and checked for specificity before use.

### STAT3 siRNA assay

Interference of STAT3 by small interfering RNA was performed as previously described [47]. Briefly, PCa cells were plated onto a 3.5-cm dish and allowed to adhere for 16 h. Cells were transiently transfected with either the recombinant STAT3-siRNA or the non-targeting scrambled siRNA in complete medium for 48 h. Cells were washed and transferred to complete medium for another 48 h and then harvested for the subsequent experiments.

### PC-3M subcutaneous xenograft animal model

PC-3M cells (1 × 10^6^ in 0.1mL PBS per mouse) were inoculated subcutaneously on the right back sides of the mice. After the tumors reached about 100 mm^3^, mice were divided into 3 groups and received i.p injection either with DMSO or Lycorine (5 mg/kg/day per mouse and 10 mg/kg/day per mouse) every day for 18 days. During the administration of Lycorine, the body weight and the tumor size of the mice were monitored every 2 days. Mice were continually observed until they were sacrificed.

### PC-3M-luc orthotopic transplantation xenograft model

Orthotopic implantation of tumor cells was performed as described previously [48]. Briefly, midline incisions were made, superior to the genital area of supine anesthetized male athymic mice (nu/nu). The bladder was externalized and extended with a cotton swab to reveal the seminal vesicles and the dorsum of the prostate. All procedures were performed in sterile environment. PC-3M-luc cells (5 × 10^5^ in 0.02 mL PBS per mouse) were orthotopically transplantated into the dorsa lope of prostate of nude mice after anaesthetized by pentobarbital sodium. Seven days later, based on photon flux indexes detected by Xenogen IVIS 2000 Luminal Imager, all mice bearing tumor were divided into three groups (*n* = 20 per group) randomly and from that day on, the luminal photos were taken and photon flux indexes which could represent the orthotopic tumor sizes were recorded every 5 days. Lycorine (5 mg/kg/day per mouse and 10 mg/kg/day per mouse) was injected intraperitoneally every day. The control group was treated with DMSO. 30 days later, mice were sacrificed, and tumors in prostates were removed and images were recorded. To assess metastasis, the residual fluorescence of multiple organs (liver, lung, kidney, spleen and bone) was measured. The growth rate curve of the tumor xenograft was evaluated by determining the photon flux indexes. The mouse body weight was measured every 2 days. Prostate tumor xenografts were fixed and prepared for immunohistochemistry.

### Immunohistochemistry analysis

Immunohistochemistry was performed as previously reported [44]. Primary tumors and mets were excised, fixed and embedded in paraffin. To investigate the effect of Lycorine on tumor cells proliferation and apoptosis *in vivo*, sections (4 μm) were stained with anti-proliferation cell nuclear antigen (Ki-67), cleaved caspase-3, p-STAT3 and E-cadherin. Images were obtained with Leica microscope (Leica, DM4000b). The results were analyzed using Image-Pro Plus 6.0 software.

### Chromatin immunoprecipitation assay

PC-3M cells were treated with various concentrations (0, 1, 5, 10, and 25 μM) of Lycorine for 48 hours, fixed with 1% formaldehyde, and lysed as described previously [45]. Chromatin samples were immunoprecipitated with antibodies against STAT3 or with normal rabbit IgG antibody and examined by quantitative PCR using the SYBR Premix Ex Taq Kit (TaKaRa Biotechnology).

### Statistical analysis

Results were statistically analyzed using the Student's *t* test with Microsoft Excel except for the survival curve and mets statistical result, which were analyzed using GraphPad Prism version 4.02 for Windows. All experiments were repeated at least three times. A value of *P* < 0.05 was considered statistically significant.

## SUPPLEMENTARY FIGURES


