# Supplementary material for: Lycorine is a novel inhibitor of the growth and metastasis of hormone-refractory prostate cancer
Source: Oncotarget. 2015 Apr 12;6(17):15348–61. doi: 10.18632/oncotarget.3610 (PMC4558156; doi:10.18632/oncotarget.3610)
Supplement: Supplementary file 1 [file oncotarget-06-15348-s001.pdf]

SUPPLEMENTARY FIGURES

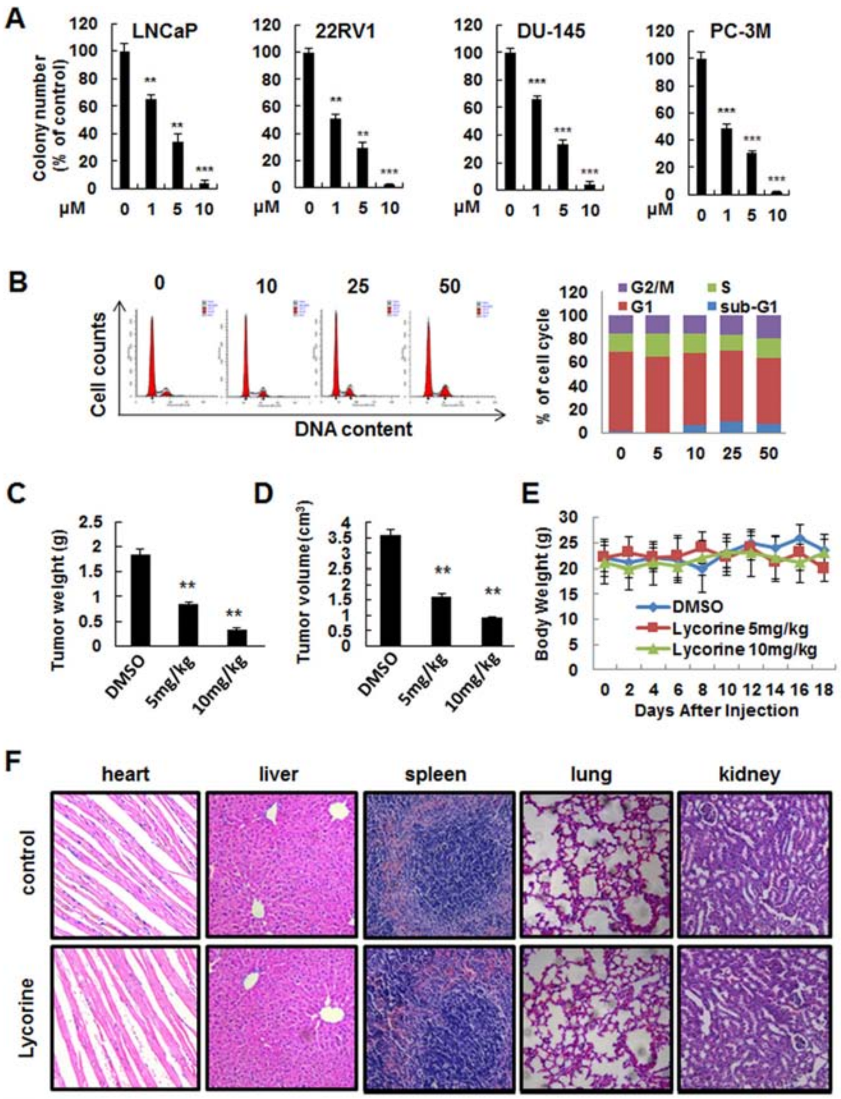

Supplementary Figure S1: *In vitro* and *in vivo* inhibitory effects of Lycorine on PCa. A. Statistic results of Fig. 2A for 4 PCa cell lines, respectively. **B.** PC-3M cells were treated with indicated concentrations of Lycorine (from 0 μM to 50 μM) for 36 hours. Cell cycle distribution was evaluated using propidium iodide (PI) staining and flow cytometry ($n = 3$) and the statistic result was measured. **C.** Statistic result of final tumor weights of 3 groups in the subcutaneous tumor model. **D.** Statistic result of final orthotropic tumor volumes of 3 groups in the orthotopic tumor model. **E.** Lycorine's effects to the body weight of mice when recorded every 2 days. **F.** H&E staining of the anatomy results of sacrificed mice main organs, including heart, liver, spleen, lung and kidney in the control group and the 10 mg/kg/day group.

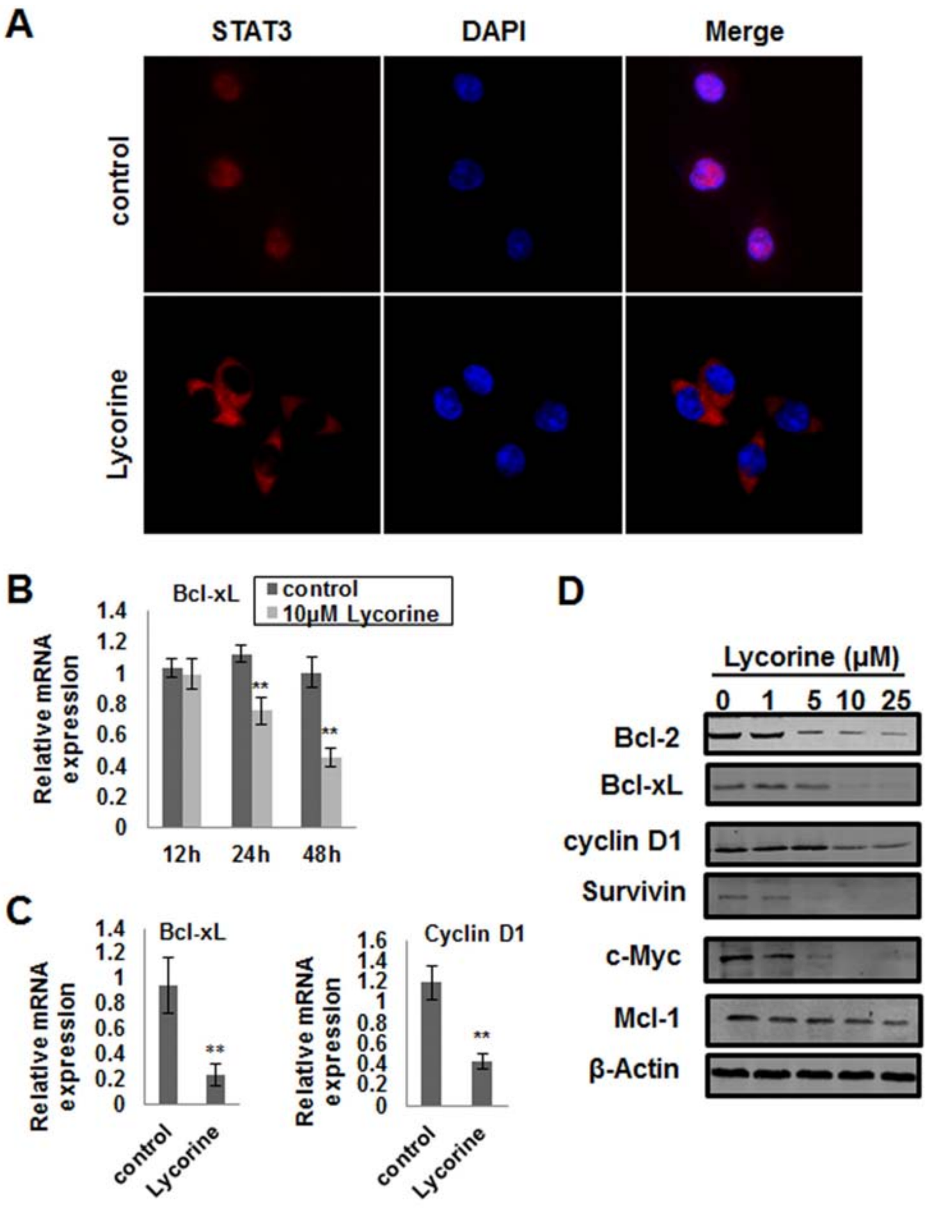

Supplementary Figure S2: Effect of Lycorine on STAT3 transcriptional activity. A. PC-3M cells were treated with 10 μM Lycorine and 10 ng/ml EGF for 24 hours. The localization of STAT3 in cytoplasm or nuclei was visualized by immunofluorescence staining as described in Material and Methods (magnification × 40). All experiments were repeated at least three times. **B.** PC-3M cells were pretreated with 10 μM Lycorine for 12 h, 24 h and 48 h, respectively, and then incubated with 10 ng/ml EGF for 4 hours. Total RNA was isolated and subjected to Q-PCR by Bcl-xL primers. **C.** PC-3M cells were treated with 10 μM Lycorine for 24 h and subjected to Q-PCR to test the transcription of Bcl-xL and cyclin D1. **D.** PC-3M cells were treated with 10 ng/ml EGF and indicated concentrations of Lycorine (from 0 μM to 25 μM) for 24 hours, then prepared for Western blotting of target proteins of STAT3 such as Bcl-2, Bcl-xL, cyclin D1, Mcl-1and survivin.
